# R2Tg retrotransposon mediated targeted integration of exogenous genes into the 28S rDNA locus in mammalian cell lines for PKU gene therapy

**DOI:** 10.3389/fgeed.2026.1893815

**Published:** 2026-09-15

**Authors:** Xiuyuan Wang, Ziqi Wen, Rongjun Xu, Wenjing Shi, Yiling Long, Xingri Zhan, Rui Su, Jia Fei, Yumin Li

**Affiliations:** 1 State Key Laboratory of Bioactive Molecules and Druggability Assessment, Department of Biochemistry and Molecular Biology, Medical College of Jinan University, Guangzhou, China; 2 Guangdong Engineering Technology Research Center of Drug Development for Small Nucleic Acids, Guangzhou, China; 3 Antisense Biopharmaceutical Technology Co., Ltd., Guangzhou, China; 4 Medical Laboratory of the Third Affiliated Hospital of Shenzhen University, Shenzhen, China

**Keywords:** 28S rDNA, phenylalanine hydroxylase (PAH) mRNA, phenylketonuria (PKU), R2 retrotransposon in *Taeniopygia guttata* (R2Tg), targeted gene integration, target-primed reverse transcription (TPRT)

## Abstract

Phenylketonuria (PKU) remains a challenging genetic disorder due to the absence of therapeutic strategies capable of achieving sustained restoration of phenylalanine hydroxylase (PAH) function. Here, we employed an R2 retrotransposon in *Taeniopygia guttata* (R2Tg)-based system to enable targeted integration of exogenous PAH mRNA into the 28S ribosomal DNA (rDNA) locus of the host genome, with the goal of rescuing PAH deficiency caused by loss-of-function mutations. Experimental results demonstrated that lipid nanoparticle (LNP) delivery of R2Tg facilitated efficient site-specific integration of EGFP and PAH transgenes in mammalian cells, significantly elevating PAH mRNA and protein expression without disrupting endogenous 28S rRNA levels. These findings support the potential of this strategy as a precise genomic modification tool with promise for lifelong correction of PAH deficiency.

## Introduction

PKU is caused by mutations in the PAH gene in the liver, leading to reduced or absent PAH activity. This impairs the conversion of phenylalanine (Phe) to tyrosine, resulting in elevated Phe levels in the blood and brain (Hillert et al., 2020). Clinical manifestations include intellectual developmental disorders, behavioral abnormalities, and hypopigmentation of skin and hair (Blau et al., 2010; Elhawary et al., 2022). Although early diagnosis and treatment through newborn screening programs in most countries enable near-normal intellectual development in PKU patients, lifelong adherence to strict dietary restrictions is still required. Furthermore, some approved medications are limited to adult use (Elhawary et al., 2022). Therefore, there is an urgent need to explore novel therapeutic approaches for PKU.

Emerging gene therapies based on site-specific genomic integration represent a promising direction for PKU treatment. In recent years, an increasing number of studies have focused on the R2tg retrotransposon technology. The endogenous R2Tg are non-long terminal repeat (non-LTR) retrotransposons that utilize an RNA intermediate and operate through a target-primed reverse transcription (TPRT) mechanism to reverse transcribe and integrate genetic material (Zhang et al., 2025; Fell et al., 2025). Therefore, after undergoing engineering modifications, R2tg has become a key tool for achieving gene writing. Compared to existing gene editing tools, the R2Tg retrotransposon offers the following advantages: Targeted integration specificity-precise localization to “safe harbor” sites, reducing off-target risks; fully RNA-mediated delivery, which lowers immunogenicity and the risk of random integration, making it more suitable for clinical applications (Chen et al., 2024).

In this study, we combined R2Tg with mRNA technology, employing it to deliver exogenous PAH mRNA for integration into the 28S rDNA locus in somatic cells. This strategy aims to supplant mutant or defective PAH copies, thereby restoring enzymatic function and offering a potential treatment for PAH-deficient disorders.

## Materials and methods

Optimized R2Tg mRNA and donor mRNAs (EGFP or PAH) were synthesized via *in vitro* transcription, featuring 5′ and 3′ UTRs from HBA1/α-globin, and were encapsulated in Lipid Nanoparticles (LNPs). 293T and HepG2 cell lines were cultured and transfected with a combination of R2Tg-LNP and either EGFP- or PAH-donor-LNPs. Genomic DNA was extracted post-transfection, and successful targeted integration into the 28S rDNA locus was detected using nested PCR with junction-specific primers. Reverse transcription-quantitative PCR (RT-qPCR) was performed on extracted RNA to assess transgene expression levels, while flow cytometry and immunofluorescence were used to analyze EGFP and PAH protein expression, respectively. Data from gel electrophoresis, qPCR, and flow cytometry were analyzed and visualized using specialized software (ImageJ, CFX Maestro, FlowJo). Statistical analyses were conducted with GraphPad Prism. Two-tailed unpaired Student’s t-tests were used for pairwise comparisons between two independent groups. F-test was performed to test homogeneity of variances prior to each t-test; standard Student’s t-test assuming equal variance was used when variances were not significantly different. Statistical significance threshold was set at P < 0.05. Data are shown as mean ± SEM. Experimental details are available in the Supplementary Material.

## Results

### Targeted genomic integration of PAH via LNP-delivered dual R2Tg/PAH mRNA system

Lipid nanoparticles (LNPs) co-deliver two synthetic RNAs encoding R2Tg transposes and PAH donor template into target cells. In the cytoplasm, R2Tg mRNA is translated into functional transposes, which specifically binds the 3′ UTR and 5′ UTR of PAH mRNA to assemble Ribonucleoprotein (RNP) complexes. The RNP complex translocates into the nucleus and initiates target-primed reverse transcription (TPRT) at the conserved 28S rDNA safe-harbor site. Following sequential single-stranded DNA cleavage, reverse transcription and host DNA repair, the exogenous PAH gene is stably integrated into the 28S rDNA locus. Integrated PAH cassettes are subsequently transcribed and translated to produce functional PAH protein (Figure 1). Extended ultra-fine molecular descriptions of R2Tg domain architecture and retro-transposition mechanisms can is available in Supplementary Figures S1, S2. To carry out the subsequent research, PAH, EGFP, and R2tg mRNAs were first synthesized by *in vitro* transcription (Supplementary Figure S3).

**FIGURE 1:**
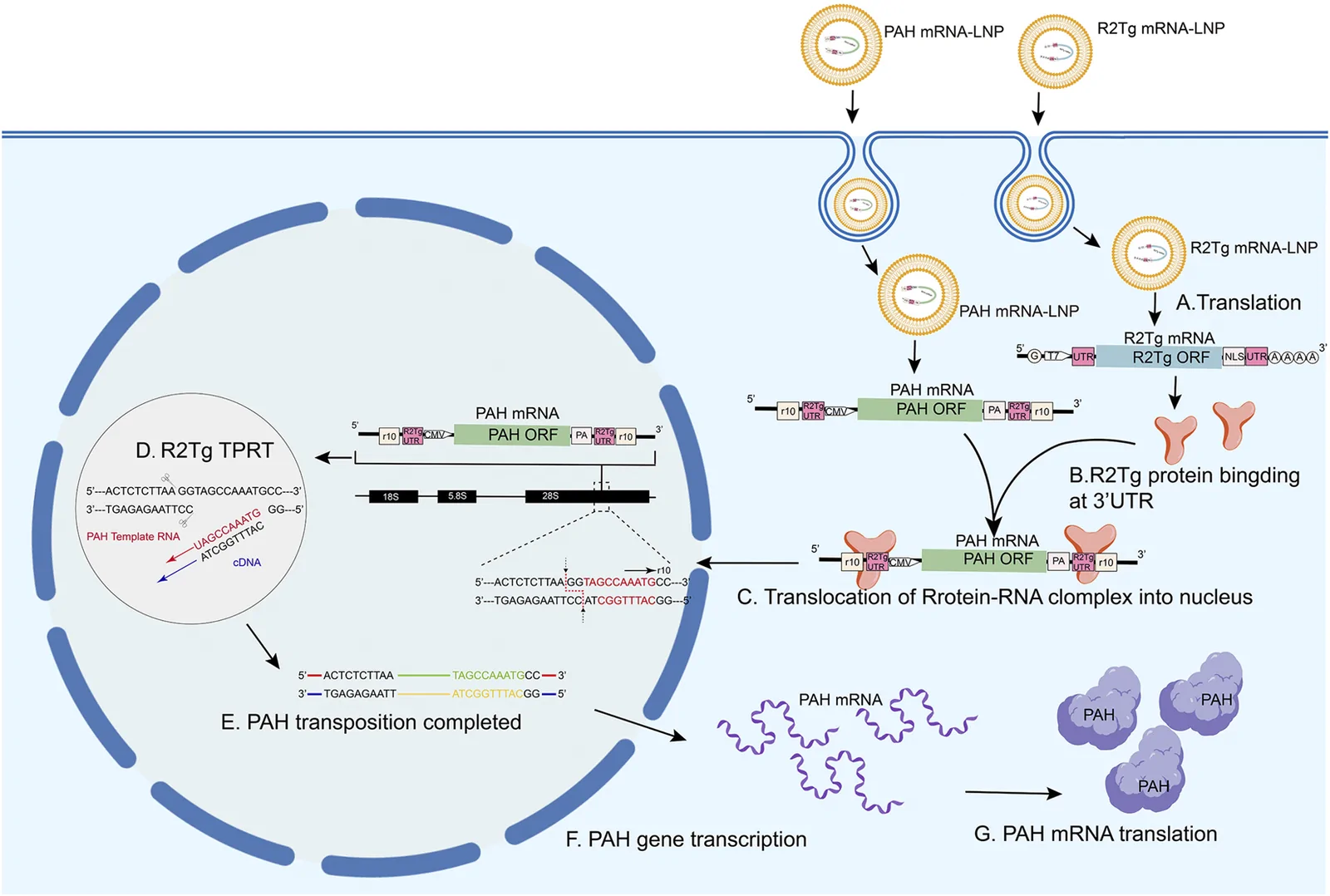
Schematic illustration of LNP-delivered dual-mRNA system for R2Tg-mediated PAH gene targeted transposition. LNPs encapsulating R2Tg RNA and PAH donor RNA are internalized by cells. Cytosolic R2Tg protein binds PAH mRNA to form RNP complexes that translocate into the nucleus. The R2Tg machinery catalyzes TPRT reactions to achieve stable insertion of the PAH gene at the 28S rDNA locus, supporting persistent PAH protein expression.

### Directed insertion and functional expression of EGFP achieved by R2Tg in mammalian cells

In order to verify that R2Tg can mediate gene insertion,we first examined the gene insertion events in 293T and HepG2 cells. Nested PCR analysis targeting the 5′ and 3′ junction sites (Figure 2A) confirmed successful insertion of the target sequence. To determine the precision of insertion events, sanger sequencing was further conducted on PCR products (Figure 2B). The results demonstrated that R2Tg efficiently ligated the 5′ and 3′ termini of EGFP cDNA to 28S rDNA, respectively (Supplementary Figure S4A). Western blot analysis (Figure 2C) further demonstrated the expression of R2Tg proteins, indicating the functional integration facilitated by R2Tg.

**FIGURE 2:**
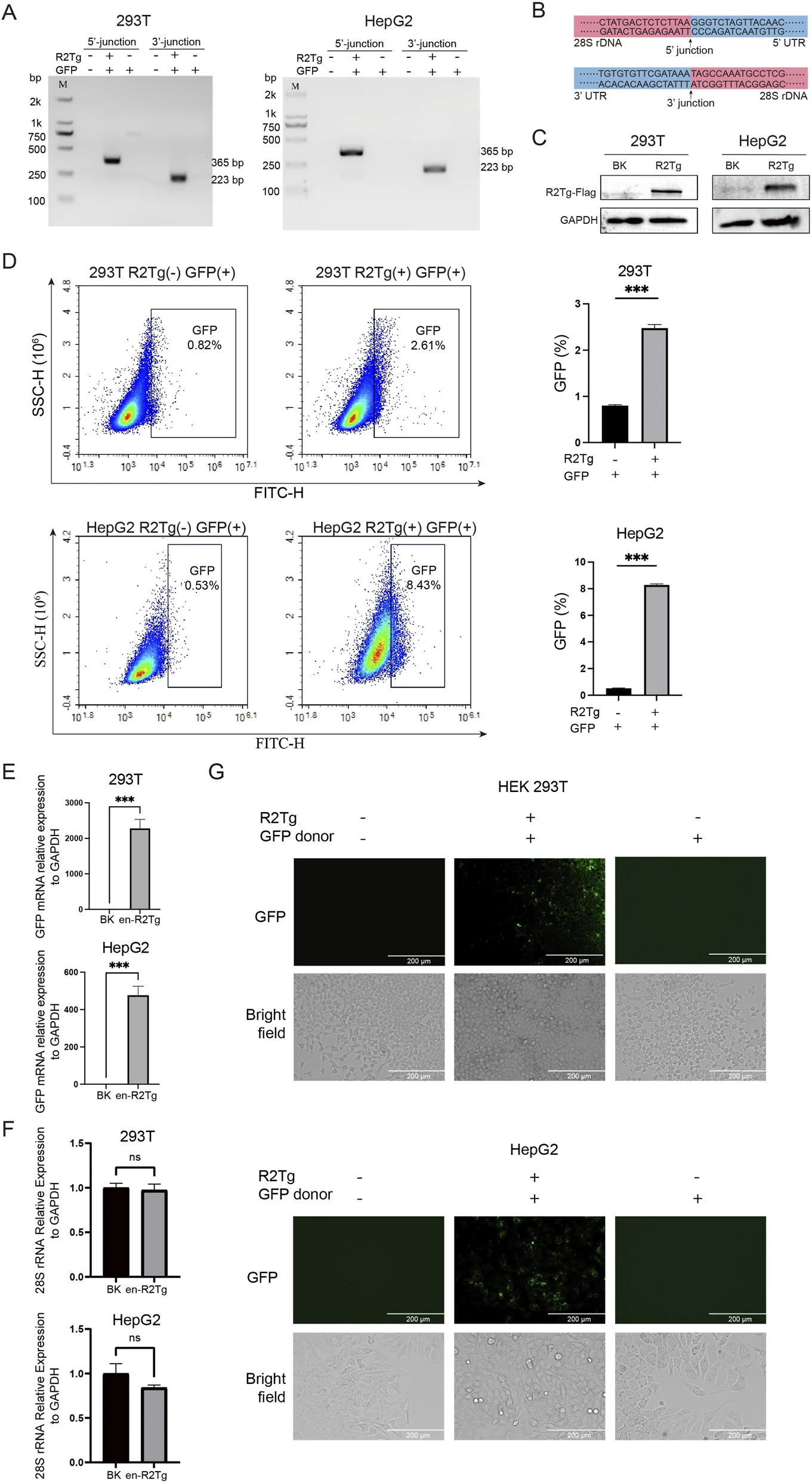
R2Tg mediates retrotransposition and functional expression of EGFP in 293T and HepG2 cells. **(A)** Nested PCR validation of R2Tg - mediated gene insertion. Genomic DNA from 293T and HepG2 cells (with/without R2Tg) was analyzed by PCR targeting 5′ - and 3′ - junctions of the inserted sequence. Bands at ∼360 bp (5′ - junction) and ∼223 bp (3′ - junction) indicate insertion, with DNA size markers (M) for reference. **(B)** Schematic representation of sequences at junctions of cDNA 5′ UTR, 3′ UTR and flanking regions surrounding the 28S rDNA cleavage site. **(C)** Western blot analysis was performed to detect R2Tg-flag expression in the blank control group (BK) and in 293T and HepG2 cells transfected with R2tg-mRNA LNP, using GAPDH as the internal control. **(D)** Flow cytometry analysis of EGFP + cells. 293T and HepG2 cells were analyzed for EGFP fluorescence. The flow cytometry dot plot shows that the EGFP+ cell population (gated region) is increased in the R2Tg and GFP co-transfection group compared with the R2Tg-only transfection group. The fluorescence intensity is 2.61% in 293T cells and 8.43% in HEPG2 cells. **(E,F)** qPCR analysis of EGFP and 28S rRNA expression, mRNA levels relative to GAPDH of EGFP **(E)** and 28S rRNA **(F)** in 293Tand HepG2 cells. **(G)** Fluorescence microscopy of EGFP expression. 293T and HepG2 cells were imaged for EGFP (green) and bright - field views. Scale bars = 200 μm. (n = 3, mean ± SD; ***p < 0.001, Student’s t-test).

Flow cytometry analysis (Figure 2D) verified the successful heterologous expression of exogenous R2Tg and EGFP in 293T and HepG2 cells; specific expression signals of R2Tg and EGFP were detected in the transfected groups, with no detectable signals in the blank controls of both cell lines. Therefore, targeted integration efficiency is defined as the proportion of EGFP^+^ viable cells gated via flow cytometry, reflecting the fraction of cells carrying site-specific transgene insertion within the 28S rDNA locus. Under the established LNP co-delivery platform, R2Tg-driven targeted integration efficiencies hit 2.61% in 293T cells and 8.43% in HepG2 hepatic cells (Figure 2D). qPCR analysis indicated the upregulation of EGFP mRNA (Figure 2E), while the 28S rRNA expression remained relatively stable (Figure 2F), indicating the specific effect on EGFP gene expression. Fluorescence microscopy (Figure 2G) visually demonstrated the enhanced EGFP expression in the presence of R2Tg, further validating our flow cytometry and qPCR results.

### R2Tg-mediated directed insertion of the PAH gene and its protein expression in 293T cells

Next, to achieve the treatment of PKU by R2Tg retrotransposon, the PAH mRNA Reverse Transcription and Expression was verified *in vitro*. For PAH mRNA, nested PCR analysis (Figure 3A) verified the R2Tg - driven gene insertion events. Furthermore, sequencing data confirmed that R2Tg achieves ligation between the 5′ and 3′ termini of PAH cDNA and the regions upstream and downstream of the 28S rDNA cleavage site (Supplementary Figure S4B). qPCR analysis (Figure 3B) confirms a significant upregulation of PAH mRNA in 293T cells with R2Tg, while 28S rRNA expression was not affected (Figure 3C). Immunofluorescence analysis (Figure 3D) showed the expression and localization of PAH protein, indicating the successful R2Tg -enabled PAH gene activation.

**FIGURE 3:**
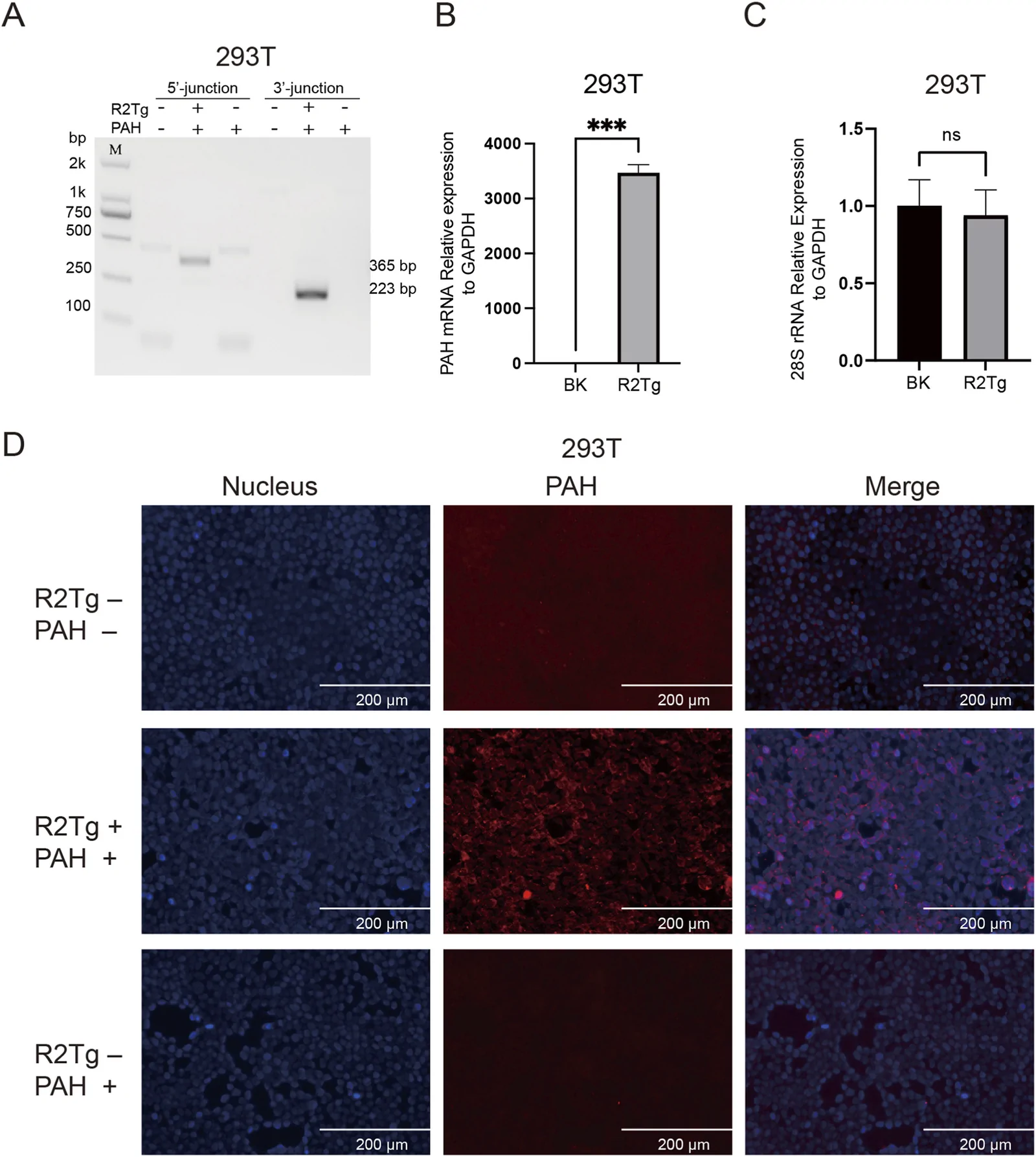
R2Tg mediates PAH mRNA retro-transposition and expression in 293T cells. **(A)** Nested PCR validation of PAH gene insertion. Genomic DNA from 293T cells (with/without R2Tg and PAH donor) was analyzed by PCR targeting 5′ - and 3′ - junctions. Bands confirm R2Tg - driven PAH insertion, with DNA size markers (M) for reference. **(B)** qPCR analysis of PAH mRNA expression. Relative PAH mRNA levels in 293T cells. **(C)** qPCR analysis of 28S rRNA expression. Relative 28S rRNA levels in 293T cells (n = 3, mean ± SD, ***p < 0.001, Student’s t-test). **(D)** Immunofluorescence analysis of PAH protein. 293T cells were stained for nuclei (blue, DAPI) and PAH protein (red). Merged images indicate PAH localization in R2Tg + PAH + groups. Scale bars = 200 μm.

## Discussion

In summary, this results clearly verified that the R2Tg retrotransposon can mediate the reverse transcription and gene insertion of both EGFP and PAH mRNAs (Figures 2, 3). This provides a novel tool for gene function research and potential gene therapy applications.

The ability of R2Tg to facilitate site-specific integration without significantly affecting housekeeping genes such as 28S rRNA is particularly noteworthy. This attribute minimizes the risk of insertional mutagenesis and off-target effects, which have been major concerns with conventional viral vectors and CRISPR-based systems (Chen et al., 2024). Moreover, the fully RNA-based delivery mechanism of R2Tg reduces immunogenicity and avoids the persistent expression of DNA-editing enzymes, thereby enhancing its suitability for clinical applications.

Targeted large transgene insertion poses challenges for mammalian genome engineering. CRISPR–Cas9 HDR enables site-specific integration yet relies on genotoxic DNA double-strand breaks and exhibits low baseline efficiency for large cargos (below 10% under unselected culture conditions (Yao et al., 2017; Leal et al., 2024). Notably, the Sleeping Beauty transposon tolerates large transgenes, with integration efficiency less susceptible to cargo size than HDR. Nevertheless, its largely random genomic integration raises risks of insertional mutagenesis and variable transgene expression (Querqu et al., 2019; Kebriaei et al., 2017). R2Tg achieves locus-specific insertion without inducing genomic DSBs, with an efficiency of approximately 8.43%. Standardized parallel assays are required in future work to benchmark these platforms.

However, several challenges remain to be addressed before clinical translation. For instance, integration efficiency remains relatively low in primary hepatocytes and *in vivo* models. Accordingly, substantial room exists for further optimization of the R2Tg system. Additionally, long-term stability of the integrated transgene and potential immune responses to repeated LNP administrations warrant thorough investigation. Future studies should also explore the scalability of this system for other monogenic diseases and assess its safety profile in more physiologically relevant models.

Immune recognition of the heterologous R2Tg protein merits consideration for repeated *in vivo* delivery. Adaptive immune responses against exogenous transposase-like proteins may eliminate modified cells and limit persistent editing. Subsequent preclinical studies will evaluate R2Tg immunogenicity and explore approaches to reduce immune reactivity.

The clinical translation of LNP-based RNA therapeutics hinges on manufacturing scalability. Microfluidic mixing technologies have substantially improved GMP reproducibility, yet batch-to-batch variability and particle-size control remain challenging during scale-up (Hou et al., 2021). Co-delivery of two distinct RNAs adds further complexity, as differences in molecular size, charge, and stability necessitate optimization of formulation parameters. While LNP manufacturing has advanced significantly, further optimization of co-encapsulation strategies will be essential for future clinical application.

Despite these challenges, the R2Tg-mediated integration system represents a promising step forward in the field of gene therapy (Luo et al., 2025). Its combination of high specificity, RNA-based delivery, and compatibility with existing mRNA technologies positions it as a versatile platform for the treatment of genetic disorders such as PKU. We believe that this strategy not only expands the strategy for genetic manipulation but also opens new avenues for achieving lifelong correction of metabolic diseases.

## Data Availability

The original contributions presented in the study are included in the article/Supplementary Material, further inquiries can be directed to the corresponding authors.
