# Supplementary material for: R2Tg retrotransposon mediated targeted integration of exogenous genes into the 28S rDNA locus in mammalian cell lines for PKU gene therapy
Source: Front Genome Ed. 2026 Sep 15;8:1893815. doi: 10.3389/fgeed.2026.1893815 (PMC13621248; doi:10.3389/fgeed.2026.1893815)
Supplement: Supplementary file 1 [file Supplementaryfile1.docx]

Supplementary Materials for

**R2Tg retrotransposon mediated targeted integration of exogenous genes into the 28S rDNA locus in mammalian cell lines for PKU gene therapy**

**This file includes:**

Supplementary Figure S1-4

Supplementary Materials and Methods

**The protein structure of R2Tg and the molecular mechanism of its retro-transposition**

The R2Tg protein comprises four functional domains: a DNA-binding domain (including N-ZnF and Myb motifs), an RNA-binding domain, a reverse transcriptase (RT) domain, and an endonuclease (EN) domain, which collectively facilitate recognition, genomic incorporation, and cleavage processes (Figure S1A and C)(1,2). The 3' end of the template PAH mRNA is integrated into the bottom strand of the 28S rDNA, enabled by recognition of the target site by the R2Tg reverse transcriptase. The N-terminal DNA-binding domain (ZNF and Myb) of the upstream-bound R2Tg reverse transcriptase detects the RUM sequence (A_–31_ACGGCGGGA_–22_), separating the two strands of the target DNA at the ZnF domain. Subsequently, a restriction-like endonuclease (RLE) active site or the EN domain cleaves the bottom strand at the RASIN site (T_–6_TAAGGT_+1_), while the upstream DNA bends by 40°, axially rotating the nick into the RT active site. The nicked bottom strand anneals with any 3' homologous strand, ultimately initiating the reverse transcription process (Figure S1B)(1).

**The principle of R2Tg-mediated insertion of exogenous PAH gene into the 28S rDNA locus**

Using liver-targeted LNP to deliver PAH mRNA and R2Tg mRNA, the R2Tg mRNA enters the cytoplasm and is translated into R2Tg protein (containing a nuclear localization signal, NLS). First, the R2Tg protein binds to the 3'UTR region of the PAH mRNA, forming a "protein-RNA complex." Then, leveraging the nuclear localization signal (NLS) of R2Tg, the complex enters the nucleus through the nuclear pore complex. Finally, the R2Tg protein recognizes and binds to the 28S rDNA site in the genome, using genomic DNA as a primer and employing TPRT to integrate the exogenous gene into the genome(Figure S2E)(3) . The R2Tg integration reaction involves the following five steps: The R2Tg protein binds to the 3' end of PAH mRNA and cleaves the bottom strand of 28S rDNA, generating a 3'OH at the nick site; R2Tg employs the broken DNA target upstream of the insertion site as a primer to catalyze reverse transcription of PAH RNA; the R2 protein bound downstream of the insertion site binds to the 5' end of PAH mRNA and cleaves the top strand of 28S rDNA; as the reverse transcription reaction catalyzed by the upstream protein displaces the 5' RNA, the downstream protein cleaves the top DNA strand and utilizes the cleaved DNA to initiate second-strand DNA synthesis while removing the PAH mRNA; the host repair mechanisms complete the integration reaction, achieving PAH gene transposition (Figure S2F)(4).

**Supplementary Figure S1:** The protein structure and domain of R2Tg protein. (A) The four domains of R2Tg protein from N- to C-terminus. Brown: DNA-binding domain; Purple: RNA-binding domain; Blue: Reverse transcriptase domain; Orange: Endonuclease domain. (B) Interaction between R2Tg protein and 28S rDNA. Protein domain positions are indicated by shaded rectangles in corresponding colors; RUM sequence is highlighted in purple; RASIN sequence is highlighted in red. (C) Domain architecture of R2Tg protein, including DNA-binding domain, RNA-binding domain, reverse transcriptase domain, and endonuclease domain. (D) Schematic structure of the template RNA, arranged from 5’to 3’: 5’ 28S rDNA homologous region, 5’ UTR, ORF (encoding PAH protein), 3’ UTR, and 3’ 28S rDNA homologous targeting region.

**Supplementary Figure S2**：Molecular mechanism of PAH mRNA retro-transposition by R2Tg. (A) After delivery of R2Tg mRNA and template mRNA into hepatocytes via LNP system, R2Tg mRNA is translated into protein in the cytoplasm and forms an RNP complex with the template mRNA. This complex enters the nucleus during mitosis and integrates into the genome during S-phase via the TPRT mechanism. (B) Steps of R2Tg integration: R2Tg protein binds to the 3’ and 5’ ends of PAH mRNA, cleaves the 28S rDNA double strand in steps, utilizes the RNA as a template to catalyze reverse transcription and second-strand synthesis, and finally completes PAH gene transposition through host cell repair mechanisms.

**Supplementary Figure S3**：Electrophoresis analysis of mRNA length after in vitro transcription (IVT), capping, and tailing. A-B show agarose gel electrophoresis results of mRNA samples prepared by in vitro transcription, followed by capping and tailing modifications. The electrophoresis was performed to verify the integrity and expected length of the in vitro transcribed mRNAs (including PAH mRNA, R2Tg mRNA, and EGFP mRNA) after capping and tailing modifications, ensuring their suitability for subsequent LNP encapsulation and cell transfection experiments.

**Supplementary Figure S4**： Sanger sequencing confirms R2Tg-directed gene integration at 28S rDNA sites. (A) Diagram of the 28S rDNA-EGFP integrated construct and Sanger sequencing chromatograms of 5′ and 3′ integration junctions obtained from 293T and HepG2 cells. (B) Schematic illustrating the 28S rDNA-PAH integrated cassette and representative Sanger sequencing traces of 5′ and 3′ junction regions in 293T cells. Blue shaded areas denote native 28S rDNA sequences.

**R2Tg^opt^ mRNA sequence:**

Introducing the CMP domain and three mutations: A793V, Q955R, and G1098R

5’UTR(HBA1)-NLS-R2Tg^opt^（CMP（N）+CMP(IL-2)）-NLS-3xFlag-3’UTR(α-globin)：

5’UTR(HBA1)

GAATTCTAATACGACTCACTATAAGGAAATAAGAGAGAAAAGAAGAGTAAGAAGAAATATAAGAGCCACC**ATG**CCCAAGAAGAAGAGGAAGGTTCCCGGGATGCCTAAGAGAAAGGTGTCCAGCGCCGAGGGCGCCGCCAAAGAAGAGCCTAAGCGGAGAAGCGCCAGACTGAGCGCCAAGCCTCCAGCTAAAGTGGAAGCCAAGCCCAAGAAGGCCGCTGCCAAGGACAAGAGCTCTGATAAGAAAGTCCAGACCAAGGGAAAACGGGGCGCTAAAGGCAAGCAGGCCGAGGTGGCCAACCAGGAGACAAAGGAAGATCTGCCTGCTGAGAACGGCGAAACCAAGACCGAGGAAAGCCCCGCCAGCGACGAGGCCGGCGAGAAGGAGGCCAAGTCTGACGCCAGCTGCCCCAAGCCAGGCCCACCTGTGTCTGCCGGCGCCATGAGCCTGGAATCTGGACTGACAACACACAGCGTGCTGGCCATCGAGAGAGGACCCAACAGCCTGGCCAATTCCGGCTCTGATTTCGGCGGCGGCGGCCTGGGCCTGCCACTAAGACTGCTGCGTGTATCAGTGGGCACGCAGACCAGCAGAAGCGATTGGGTGGATCTGGTGAGCTGGTCCCACCCTGGCCCTACAAGCAAGTCCCAGCAGGTTGACCTTGTGAGCCTGTTCCCGAAGCACCGGGTGGACCTGCTATCCAAAAATGATCAGGTGGATCTGGTCGCGCAATTCCTGCCTTCAAAGTTCCCTCCCAACCTGGCTGAAAATGACCTCGCCCTGCTGGTGAACCTGGAATTTTACAGAAGCGACCTGCACGTGTACGAATGCGTGCACTTCGCTGCCCATTGGGAGGGCCTGAGCGGACTGCCAGAAGTCTACGAACAGCTCGCCCCTCAGCCTTGCGTGGGAGAGACCCTGCATAGCTCCCTGCCTCGGGATAGCGAGCTGTTCGTGCCTGAGGAGGGCTCTAGCGAAAAGGAATCGGAAGATGCCCCCAAGACCAGCCCCCCCACCCCTGGCAAACATGGCCTGGAGCAGACAGGCGAGGAGAAGGTGATGGTTACCGTCCCCGACAAGAACCCTCCCTGTCCTTGCTGCGGCACCAGAGTGAACAGCGTGCTGAATCTGATCGAGCACCTGAAGGTGAGCCACGGCAAGCGTGGAGTTTGTTTCCGGTGCGCCAAGTGCGGCAAGGAAAACAGCAACTACCACAGCGTGGTGTGCCACTTTCCTAAGTGTAGAGGCCCTGAGACAGAAAAGGCCCCTGCCGGAGAATGGATTTGTGAGGTGTGCAACCGGGATTTTACCACCAAGATCGGCCTGGGCCAGCACAAACGGCTGGCCCACCCCGCCGTGAGAAACCAGGAGAGGATCGTGGCCAGCCAACCTAAAGAGACAAGCAACAGAGGCGCCCACAAGCGGTGCTGGACGAAGGAAGAGGAAGAATTGTTGATCAGACTGGAAGCCCAGTTCGAGGGAAACAAGAACATCAACAAGCTGATTGCCGAGCACATCACCACCAAGACCGCCAAGCAGATCAGCGACAAGAGAAGGCTGTTAAGCAGAAAGCCTGCCGAGGAGCCCAGAGAGGAACCTGGCACCTGCCACCACACCCGGAGAGCCGCCGCTTCACTGCGGACCGAGCCCGAGATGTCTCACCACGCCCAGGCCGAGGACAGAGATAACGGCGGAGGAATGCCTAAGAGAAAGGTGTCCAGCGCCGAGGGCGCCGCCAAAGAAGAGCCTAAGCGGAGAAGCGCCAGACTGAGCGCCAAGCCTCCAGCTAAAGTGGAAGCCAAGCCCAAGAAGGCCGCTGCCAAGGACAAGAGCTCTGATAAGAAAGTCCAGACCAAGGGAAAACGGGGCGCTAAAGGCAAGCAGGCCGAGGTGGCCAACCAGGAGACAAAGGAAGATCTGCCTGCTGAGAACGGCGAAACCAAGACCGAGGAAAGCCCCGCCAGCGACGAGGCCGGCGAGAAGGAGGCCAAGTCTGACGGAGGACCCGGCAGACGGCCTCTCCCCGGAAGGGCTGCCGCCGGCGGAAGAACCATGGATGAGATCAGAAGACACCCCGACAAAGGAAATGGCCAGCAGCGGCCTACCAAGCAGAAATCCGAAGAACAGCTGCAGGCTTACTACAAGAAGACCCTGGAAGAGAGACTGAGCGCCGGAGCCCTGAACACCTTTCCTAGAGCCTTCAAACAGGTGATGGAAGGCCGGGACATAAAGCTGGTCATCAACCAGACAGCCCAGGATTGCTTCGGCTGTCTGGAGAGCATCTCCCAGATTAGGACCGCTACACGGGATAAGAAGGATACCGTGACAAGAGAGAAACACCCTAAGAAGCCCTTCCAGAAGTGGATGAAAGACAGAGCAATCAAGAAAGGCAATTACCTGAGATTCCAGAGACTGTTTTACCTGGACAGAGGCAAGCTGGCCAAAATCATTCTGGACGACATCGAGTGTCTGAGTTGCGACATCCCTCTGTCCGAGATCTACAGCGTGTTCAAAACTCGGTGGGAAACAACAGGCAGCTTCAAGTCTCTGGGCGACTTCAAGACATACGGCAAAGCCGATAACACGGCTTTTAGAGAGCTGATCACCGCCAAGGAGATTGAAAAGAACGTGCAGGAGATGAGCAAGGGCAGCGCCCCTGGCCCCGATGGCATCACCCTGGGAGATGTGGTGAAGATGGACCCTGAGTTCAGCAGAACGATGGAGATTTTCAATCTGTGGCTGACCACCGGCAAGATTCCCGACATGGTGCGGGGATGTCGGACCGTGCTGATCCCAAAGAGCTCCAAGCCCGATAGACTGAAGGACATCAACAACTGGAGACCCATCACCATCGGCAGCATCCTACTCAGACTGTTTAGCAGAATCGTGACCGCCAGACTGTCCAAGGCTTGTCCTCTGAATCCTAGGCAGAGAGGCTTCATCCGGGCTGCTGGTTGCAGCGAGAACCTGAAGCTGTTGCAGACCATCATCTGGAGCGCCAAGAGAGAGCACAGACCTCTGGGCGTGGTGTTTGTGGACATCGCCAAAGCCTTCGACACCGTGTCCCACCAGCATATCATCCACGTGCTGCAACAAAGAGAGGTGGACCCCCACATCGTGGGCCTGGTCAGCAACATGTACGAGAACATCAGCACCTACATCACAACCAAGCGGAACACCCACACCGACAAGATCCAGATCCGGGTGGGCGTGAAGCAGGGCGATCCTATGTCCCCTCTTCTGTTCAACCTGGCCATGGACCCCCTGTTGTGCAAGCTGGAGGAGAGCGGCAAGGGCTACCACCGGGGCCAGAGCAGCATCACAGCCATGGCGTTCGCCGACGATTTGGTGTTGCTGAGCGATTCTTGGGAGAACATGAACACCAACATCAGCATCCTGGAGACCTTCTGCAACCTGACCGGCCTGAAAACCCAGGGCCAGAAGTGCCACGGCTTCTATATCAAGCCTACAAAAGACAGCTACACCATCAATGACTGCGCCGCCTGGACCATCAACGGCACACCTCTGAATATGATTGACCCTGGCGAAAGCGAGAAGTACCTGGGCTTGAGATTCGACCCTTGGATCGGAATCGCCAGAAGCGGCCTGAGCACCAAGCTCGACTTCTGGTTGCAGAGAATCGACCAGGCACCACTGAAGCCCCTGCAAAAGACAGACATCCTGAAGACATACACCATCCCTCGCCTAATCTATATCGCCGACCACTCTGAGGTGAAAACCGCCCTGCTGGAAACCCTGGACCAAAAGATCCGGACAGCCGTGAAGGAATGGCTGCACCTGCCGCCTTGTACATGCGACGCTATCCTGTACAGCAGCACCAGAGATGGCGGGCTGGGCATCACTAAGCTGGCCGGCCTCATTCCTAGCGTGCAGGCCAGACGGCTGCATAGAATCGCACAAAGCTCTGATGACACCATGAAGTGCTTTATGGAGAAAGAGAAAATGGAACAGCTGCACAAGAAGCTGTGGATTCAGGCCAGAGGAGACCGAGAAAACATCCCATCTATCTGGGAGGCTCCTCCTAGCTCTGAGCCTCCTAACAACGTGTCTACAAACAGCGAGTGGGAGGCCCCTACCCAGAAGGACAAGTTCCCCAAGCCATGTAACTGGCGGAAAAACGAGTTCAAGAAGTGGACCAAGCTGGCCAGCCAGGGCCGGGGCATTGTGAACTTCGAGCGGGACAAGATCTCTAACCACTGGATTCAGTACTATAGAAGAATCCCTCACCGGAAGCTGCTGACCGCCCTGCAGCTGAGAGCTAATGTATACCCCACAAGAGAGTTCCTGGCCAGAGGAAGACAGGACCAGTACATCAAAGCCTGCAGACACTGCGATGCTGACATCGAAAGCTGTGCACATATCATCGGCAACTGCCCCGTGACCCAGGACGCCCGCATAAAGCGTCACAACTATATCTGCGAGCTTCTGCTGGAAGAAGCCAAAAAGAAAGATTGGGTGGTGTTTAAGGAACCTCACATCCGGGACAGCAACAAGGAGCTGTACAAGCCTGATCTGATCTTCGTCAAGGACGCCAGAGCTCTGGTTGTGGACGTGACTGTGAGATACGAGGCCGCTAAGTCCTCCCTGGAAGAGGCCGCCGCCGAGAAGGTGAGAAAGTACAAACACCTGGAAACCGAGGTGAGACACCTGACCAACGCCAAAGACGTGACCTTCGTCGGTTTCCCTCTGGGCGCTCGCGGCAAGTGGCACCAGGACAACTTCAAGCTCCTGACTGAACTGGGACTGTCTAAAAGCAGACAGGTGAAGATGGCAGAGACATTTTCCACCGTGGCTCTGTTCTCCTCCGTGGACATCGTCCACATGTTCGCCAGCAGAGCCCGAAAAAGCATGGTGATGGCTAGCCCAAAGAAGAAGAGGAAAGTCGGATCCGACTACAAAGACCATGACGGTGATTATAAAGATCATGACATCGATTACAAGGATGACGATGACAAGTAATAAGCTGCCTTCTGCGGGGCTTGCCTTCTGGCCATGCCCTTCTTCTCTCCCTTGCACCTGTACCTCTTGGTCTTTGAATAAAGCCTGAGTAGGAAGAAGCTTGGCGTAATCATG

R2Tg^opt^

R2Tg^opt^

SV40 NLS

CMP（N）

Kozak

CMP（IL-2）

A793V

Q955R

G1098R

R2Tg^opt^

SV40 NLS

3*FLAG

3’UTR(α-globin)

**EGFP Donor^opt^ mRNA sequence:**

5’UTR(HBA1)-LHA-5’UTR-HSV_TKpA-**GFP(Inverted)**-pCMV-3’UTR(donor^opt^)-RHA-3’UTR(α-globin):

5’UTR

GAATTCTAATACGACTCACTATAAGGAAATAAGAGAGAAAAGAAGAGTAAGAAGAAATATAAGAGTTGACGCGATGTGATTTCTGCCCAGTGCTCTGAATGTCAAAGTGAAGAAATTCAATGAAGCGCGGGTAAACGGCGGGAGTAACTATGACTCTCTTAAGGGTCTAGTTACAACTGGGCATCGCTGCAGAGATCGCACCTCCTCGTGGTCCCGCTGGTAGCCCTTCGAAGGGTGACTAAGTCGATCTCTGCCCCAGGTACGGAGCCGTTGGGACTCACCAGTCCAACGTAACTCCTGCCTAAATTCGGTGAAACAAATTCCTCGGTAAAAAGCCCCGGTACCGAACAAACGACCCAACACCCGTGCGTTTTATTCTGTCTTTTTATTGCCGATCCCCTCAGAAGAACTCGTCAAGAAGGCGATAGAAGGCGATGCGCTGCGAATCGGGAGCGGCGATACCGTAAAGCACGAGGAAGCGGTCAGCCCATTCGCCGCCAAGCTCTTCAGCAATATCACGGGTAGCCAACGCTATGTCCTGATAGCGGTCGGCCGCTTTACTTGTACAGCTCGTCCATGCCGAGAGTGATCCCGGCGGCGGTCACGAACTCCAGCAGGACCATGTGATCGCGCTTCTCGTTGGGGTCTTTGCTCAGGGCGGACTGGGTGCTCAGGTAGTGGTTGTCGGGCAGCAGCACGGGGCCGTCGCCGATGGGGGTGTTCTGCTGGTAGTGGTCGGCCAGCTGCACGCTGCCGTCCTCGATGTTGTGGCGGATCTTGAAGTTCACCTTGATGCCGTTCTTCTGCTTGTCGGCCATGATATAGACGTTGTGGCTGTTGTAGTTGTACTCCAGCTTGTGCCCCAGGATGTTGCCGTCCTCCTTGAAGTCGATGCCCTTCAGCTCGATGCGGTTCACCAGGGTGTCGCCCTCGAACTTCACCTCGGCGCGGGTCTTGTAGTTGCCGTCGTCCTTGAAGAAGATGGTGCGCTCCTGGACGTAGCCTTCGGGCATGGCGGACTTGAAGAAGTCGTGCTGCTTCATGTGGTCGGGGTAGCGGCTGAAGCACTGCACGCCGTAGGTCAGGGTGGTCACGAGGGTGGGCCAGGGCACGGGCAGCTTGCCGGTGGTGCAGATGAACTTCAGGGTCAGCTTGCCGTAGGTGGCATCGCCCTCGCCCTCGCCGGACACGCTGAACTTGTGGCCGTTTACGTCGCCGTCCAGCTCGACCAGGATGGGCACCACCCCGGTGAACAGCTCCTCGCCCTTGCTCACCATGGTGGCGAATTCGAAGCTTGAGCTCGAGATCTGAGTCCGGTAGCGCTAGCGGATCTGACGGTTCACTAAACCAGCTCTGCTTATATAGACCTCCCACCGTACACGCCTACCGCCCATTTGCGTCAATGGGGCGGAGTTGTTACGACATTTTGGAAAGTCCCGTTGATTTTGGTGCCAAAACAAACTCCCATTGACGTCAATGGGGTGGAGACTTGGAAATCCCCGTGAGTCAAACCGCTATCCACGCCCATTGATGTACTGCCAAAACCGCATCACCATGGTAATAGCGATGACTAATACGTAGATGTACTGCCAAGTAGGAAAGTCCCATAAGGTCATGTACTGGGCATAATGCCAGGCGGGCCATTTACCGTCATTGACGTCAATAGGGGGCGTACTTGGCATATGATACACTTGATGTACTGCCAAGTGGGCAGTTTACCGTAAATACTCCACCCATTGACGTCAATGGAAAGTCCCTATTGGCGTTACTATGGGAACATACGTCATTATTGACGTCAATGGGCGGGGGTCGTTGGGCGGTCAGCCAGGCGGGCCATTTACCGTAAGTTATGTAACGCCCGGGCACGGGATTTGTCCAAGGTGGACGGGCCACCTTTACTTAACCCGGAAAAGGAACATATATAATTTATGTGTGTTCGATAAATAGCCAAATGCCTCGTCATCTAATTAGTGACGCGCATGAATGGATGAACGAGATTCCCACTGTCCCTACCTACTATCCAGCGAAACCACAGCCAAGGGAAAGCTGCCTTCTGCGGGGCTTGCCTTCTGGCCATGCCCTTCTTCTCTCCCTTGCACCTGTACCTCTTGGTCTTTGAATAAAGCCTGAGTAGGAAGAAGCTTGGCGTAATCATG

LHA

HSV_TKpA

Kozak

CMV promoter

3’UTR(donor^opt^)

RHA

3’UTR(α-globin)

**PAH Donor^opt^ mRNA sequence:**

5’UTR(HBA1)-LHA-5’UTR-HSV_TKpA-**PAH(Inverted)**-pCMV-3’UTR(donor^opt^)-RHA-3’UTR(α-globin):

GAATTCTAATACGACTCACTATAAGGAAATAAGAGAGAAAAGAAGAGTAAGAAGAAATATAAGAGTTGACGCGATGTGATTTCTGCCCAGTGCTCTGAATGTCAAAGTGAAGAAATTCAATGAAGCGCGGGTAAACGGCGGGAGTAACTATGACTCTCTTAAGGGTCTAGTTACAACTGGGCATCGCTGCAGAGATCGCACCTCCTCGTGGTCCCGCTGGTAGCCCTTCGAAGGGTGACTAAGTCGATCTCTGCCCCAGGTACGGAGCCGTTGGGACTCACCAGTCCAACGTAACTCCTGCCTAAATTCGGTGAAACAAATTCCTCGGTAAAAAGCCCCGGTACCGAACAAACGACCCAACACCCGTGCGTTTTATTCTGTCTTTTTATTGCCGATCCCCTCAGAAGAACTCGTCAAGAAGGCGATAGAAGGCGATGCGCTGCGAATCGGGAGCGGCGATACCGTAAAGCACGAGGAAGCGGTCAGCCCATTCGCCGCCAAGCTCTTCAGCAATATCACGGGTAGCCAACGCTATGTCCTGATAGCGGTCGGCCGCTTTACTTTATTTTCTGGAGGGCACTGCAAAGGATTCCAATTTCACTGTTAATGGAATCAGCCAAAATCTTAAGCTGCTGGGTATTGTCCAAGACCTCAATCCTTTGGGTGTATGGGTCGTAGCGAACTGAGAAGGGCCGAGGTATTGTGGCAGCAAAGTTCCTTACTTTCTCCTTGGCATCATTAAAACTCTCTGCCACGTAATAGAGGGGCTGGAACTCCGTGACAGTGTAATTTTGGATGGCTGTCTTCTCCAGCTCCAGGGGGAGAAGCTTTGGCTTCTCTGATAAGCAGTACTGTAATTCACCAAAGGATGACAGGAGCCCAGCACCATATGCCTTTATGGAGTCTCCTTGTTTGCAGAGCCCAAACTCCACAGTAAACCAGTAAATTGTGGCGAGCTTTTCAATGTATTCATCAGGTGCACCCAGAGAGGCAAGGCCAATTTCCTGGGAAAACTGGGCAAAGCTGCGATCTGAAAACAAGGGCACATGTCCCAACAGCTCATGGCAGATGTCAGGTTCGGGGGTATACATGGGCTTGGATCCATGTCTGATGTACTGTGTGCAGTGGAAGACTCGGAAGGCCAGGCCACCCAAGAAATCCCGAGAGGAAAGCAGGCCAGCCACAGGTCGGAGGCGGAAACCAGTGCAAGTCTGCAGGAACTGAGAAACGTCTTCCAGCTGGGGAATGTTATCTTCATGGAAGCCACAGTACTTTTCAAGAAGTGGAAAAATGTGATTGTACTCATAGCAAGCATGGGTTTTATACAAGGACTTCAGAGTCTTGAACACTGTGCCCCATGTTTTCTTTTCTTCCTCCATGTATTCCACTCGAGGGATGGGCTGCCCATGGCGGTAGTTGTAGGCAATGTCAGCAAACTGCTTCCGTCTTGCACGGTACACAGGATCTTTAAAACCAGGGTGGTCAGCATCCAGTTCCGCTCCATAGCTGAGAATCTGATTGGCAAATCTGTCCAGCTCTTGAATGGTTCTTGGGAACCAGGGCACTGTGTCTTTCTTCTTATCTCGTGAAAGCTCATGGACAGTGGCACCAATGTCATGCCTCAAGATCTTGATGATGTTTGTCAGAGCAGGCAGGCTACGTTTATCCAAATGGGTGAAAAATTCATACTCATCTTTCTTTAAACGAGAAGGTCTAGATTCAATGTGGGTCAGGTTTACATCATTCTCCTCAAATAAGCGCAATACTTTGGCCAATGCACCAACTTCTTCTTTGAGTGAGAAGATCAGTGATATGGCACCATTTTGATTGCAGTTGTCTTCAATATAGCTTGTTTCCTGTCCAAAGTCAGAGAGTTTCCTGCCCAAGCCTGGGTTTTCCAGGACCGCAGTGGACATGGTGGCGAATTCGAAGCTTGAGCTCGAGATCTGAGTCCGGTAGCGCTAGCGGATCTGACGGTTCACTAAACCAGCTCTGCTTATATAGACCTCCCACCGTACACGCCTACCGCCCATTTGCGTCAATGGGGCGGAGTTGTTACGACATTTTGGAAAGTCCCGTTGATTTTGGTGCCAAAACAAACTCCCATTGACGTCAATGGGGTGGAGACTTGGAAATCCCCGTGAGTCAAACCGCTATCCACGCCCATTGATGTACTGCCAAAACCGCATCACCATGGTAATAGCGATGACTAATACGTAGATGTACTGCCAAGTAGGAAAGTCCCATAAGGTCATGTACTGGGCATAATGCCAGGCGGGCCATTTACCGTCATTGACGTCAATAGGGGGCGTACTTGGCATATGATACACTTGATGTACTGCCAAGTGGGCAGTTTACCGTAAATACTCCACCCATTGACGTCAATGGAAAGTCCCTATTGGCGTTACTATGGGAACATACGTCATTATTGACGTCAATGGGCGGGGGTCGTTGGGCGGTCAGCCAGGCGGGCCATTTACCGTAAGTTATGTAACGCCCGGGCACGGGATTTGTCCAAGGTGGACGGGCCACCTTTACTTAACCCGGAAAAGGAACATATATAATTTATGTGTGTTCGATAAATAGCCAAATGCCTCGTCATCTAATTAGTGACGCGCATGAATGGATGAACGAGATTCCCACTGTCCCTACCTACTATCCAGCGAAACCACAGCCAAGGGAAAGCTGCCTTCTGCGGGGCTTGCCTTCTGGCCATGCCCTTCTTCTCTCCCTTGCACCTGTACCTCTTGGTCTTTGAATAAAGCCTGAGTAGGAAGAAGCTTGGCGTAATCATG

PAH

HSV_TKpA

5’UTR

LHA

Kozak

CMV promoter

3’UTR(donor^opt^)

RHA

3’UTR(α-globin)

**Supplementary Materials and Methods**

**Cell Culture:** The cell lines used in this experiment included 293T and human hepatocyte HepG2. 293T and HepG2 cells were cultured in RPMI medium (Gibco BRL) supplemented with 10% FBS, 2 mM L-glutamine, 100 μg/ml streptomycin, and 100 U/mL penicillin, and incubated at 37°C under 5% CO₂.

**mRNA synthesis:** mRNA was synthesized from linearized plasmid DNA templates (R2Tg-mRNA, EGFP-mRNA and AH-mRNA) using the T7 High Yield RNA Synthesis Kit (vazyme, DD4201) following the manufacturer’s instructions. Briefly, all kit components were taken out from -20 °C, thawed on ice, vortexed for mixing, and briefly centrifuged to collect the liquid at the bottom of the tubes. The IVT reaction system was prepared on ice, and the transcription reaction was incubated at 37 °C for 2 h. After the completion of IVT, DNase I was added to the reaction mixture for 15 min to digest the residual DNA template. Subsequently, the obtained crude mRNA was purified using RNA Cleaning Beads according to the kit protocol to obtain high-purity R2Tg-mRNA and EGFP-mRNA, which were dissolved in acidic aqueous buffer for subsequent encapsulation.

**Preparation of SM-102-LNP and mRNA encapsulation:**

1. Preparation of lipid-ethanol organic phase

Lipid components (SM-102, DSPC, cholesterol, PEG2000-DMG) were accurately weighed and dissolved in anhydrous ethanol at a predetermined molar ratio (50:10:38.5:1.5) to prepare the lipid-ethanol solution, with the final lipid concentration configured as the experimental required concentration (8 mM, 12 mM or 16 mM). The lipid-ethanol solution was prepared fresh before use to avoid lipid oxidation.

2. Microfluidic encapsulation of mRNA-LNP

mRNA-LNP complexes (R2Tg-mRNA-LNP and EGFP-mRNA-LNP) were prepared via the microfluidic mixing method. The high-purity mRNA acidic aqueous solution (water phase) and the prepared lipid-ethanol solution (organic phase) were separately transferred into RNase-free syringes, with air bubbles completely removed. The syringes were connected to the NanoAssemblr microfluidic device, and the operational parameters were set as follows: the nitrogen/phosphorus (N/P) ratio was 6, the flow rate ratio of water phase to organic phase was 3:1, and the total flow rate and waste volume were adjusted according to the experimental scale. The two phases were pumped into the microfluidic chip for rapid and homogeneous mixing to form LNP-mRNA nanoparticles, and the mixed solution was collected in a sterile collection tube.

3. Post-treatment of mRNA-LNP complexes

The collected mRNA-LNP mixture was subjected to dialysis against PBS buffer to remove residual ethanol, with buffer exchange performed to ensure complete ethanol removal. The final R2Tg-mRNA-LNP and EGFP-mRNA-LNP complexes were preserved in PBS buffer for subsequent experiments.

**LNP mRNA Transfection:** 293T and HepG2 cells in the logarithmic growth phase were collected. Cells from each group were washed once with PBS and centrifuged at 800 rpm for 3 min. The cell pellet was resuspended in 1 mL of complete medium (serum-free medium not required), counted, and diluted to a suspension of 2 × 10⁵ cells/mL. Two milliliters of the suspension were added per well of a 6-well plate and cultured overnight until adherent. LNP-encapsulated mRNA drugs were removed and mixed gently. A total RNA mixture of either EGFP mRNA-LNP (2000 ng) or PAH mRNA-LNP (2000 ng) plus R2Tg-LNP (1000 ng) was added to the culture medium containing the seeded cells to achieve a final volume of 1 mL. After 6 hours of transfection, DMEM medium containing 20% FBS was added to restore the culture system to 10% FBS. Cells were cultured for an additional 3 days.

**DNA Extraction and Nested PCR:** Three days post-transfection, cells were collected. Genomic DNA was extracted using a Fast DNA Extraction Kit (Vazyme, DC112-01). DNA concentration was quantified using a Nanodrop 2000 (Thermo Scientific) for PCR assays. Primers were designed to target the heterologous UTR and the PAH ORF regions. PCR reaction mixtures were prepared on ice according to the manufacturer's instructions (Transgen, AP221-01).

PCR Primers:

5' Junction (426 bp):

5'-F: CCAGGGGAATCCGACTGTTTA

5'-R: CGCCTTCTATCGCCTTCTTG

5' Junction (365 bp):

5'-F2: CCAGGGGAATCCGACTGTTTA

5'-R2: ATAAAACGCACGGGTGTTGG

3' Junction (478 bp):

3'-F: GGCCACCTTTACTTAACCCGGA

3'-R: TGTACCGCCCCAGTCAAACTC

3' Junction (223 bp):

R2-ddPCR-F: GCCACCTTTACTTAACCCGGA

R2-ddPCR-R: CTTCACCGTGCCAGACTAGA

**Agarose Gel Electrophoresis and Imaging:** PCR products were mixed with 6× DNA Loading Buffer (Transgen, GH101-01) and carefully loaded into the wells of an agarose gel. The power supply was connected, and the voltage was set to 100 V. Samples were electrophoresed from the cathode (negative) to the anode (positive) for 30 minutes. After electrophoresis, results were visualized and documented using a gel imaging analysis system (Uvitec).

**RNA Extraction:** Culture medium was removed, and cells were washed with PBS. One milliliter of Trizol reagent was added to lyse cells, followed by incubation at room temperature for 5 minutes. Chloroform was added, the mixture was vortexed vigorously for 15 seconds, and then incubated at room temperature for 3 minutes. The mixture was centrifuged at 12,000 × g for 15 minutes at 4°C. The upper aqueous phase was carefully transferred to a new tube. An equal volume of isopropanol was added, mixed, and incubated at room temperature for 10 minutes. The sample was then centrifuged at 12,000 rpm for 10 minutes at 4°C. The isopropanol supernatant was discarded, and the RNA pellet was washed with 75% ethanol, gently vortexed, and centrifuged at 8,000 rpm for 5 minutes at 4°C. The ethanol was discarded, and the RNA pellet was air-dried at room temperature for 5-10 minutes. The RNA pellet was dissolved in DEPC-treated water and stored at -20°C or -80°C for subsequent use. RNA concentration was quantified using a Nanodrop 2000 (Thermo Scientific) for RT-PCR detection.

**RT-PCR:** Reverse transcription of RNA samples was performed using the EasyScript® One-Step gDNA Removal and cDNA Synthesis SuperMix (Transgen, AE311-02). cDNA was mixed with qPCR primers using the PerfectStart® Visual Green qPCR SuperMix kit (Transgen, AQ621-01). RT-PCR detection was carried out on a fluorescence quantitative PCR instrument (Bio-Rad, CFX Connect).

RT-PCR Primers:

EGFP-qPCR-F: CACAAGTTCAGCGTGTCCG

EGFP-qPCR-R: CTCGATGCGGTTCACCAG

28S rRNA primers:

28s-rRNA-qPCR-F: GCCTCACGATCCTTCTGACC

28s-rRNA-qPCR-R: GTGGGTGAACAATCCAACGC

GAPDH primers:

GAPDH-qPCR-F: CTGACTTCAACAGCGACACCCAC

GAPDH-qPCR-R: TGTAGCCAAATTCGTTGTCATACCAG

**Sanger sequencing:** Genomic DNA was extracted from 293T and HepG2 cells 72 h following LNP-mRNA transfection using a genomic DNA isolation kit in accordance with the manufacturer’s instructions. Fragments spanning the 5′ and 3′ integration junctions between exogenous cDNA and endogenous 28S rDNA were amplified via nested PCR. Primers targeting sequences within integrated transgenes and flanking 28S rDNA were applied for first-round and second-round amplification. PCR products were purified using a DNA purification kit to remove nonspecific amplicons and primer dimers. Purified DNA fragments were subjected to bidirectional Sanger sequencing. Sequence chromatograms were aligned against reference sequences of 28S rDNA, EGFP and PAH cDNA to characterize sequences at the junction regions and verify targeted ligation between transgene termini and 28S rDNA cleavage flanks.

**Flow Cytometry Analysis:** After instrument initialization (~7 min) and a prime wash, data acquisition was set for 100,000 events. Parameters FSC and SSC were selected. EGFP was detected in the FITC channel at medium speed. PI dye was used to exclude dead cells and detected in the PerCP channel. Thresholds were set: FSC Height -1000, SSC -10000. The file was initially saved (.ncf). Within the software, a new sample (Specimen 1) was created for the selected well on the right sidebar (96-well plate layout). Staining buffer was run first to exclude inherent debris/particles and adjust thresholds (FSC -10000, SSC -10000). For the Blank Control (BK): After starting sample acquisition, monitor the event count and sample volume processed. If the target event count (100,000) is reached before 1000 µl is processed, the acquisition volume can be reduced or the target event count increased. For the BK group: Select one well. After basic gating is applied to this well, use it as a template. Add new specimen labels, copy the gating strategy to them, rename the specimens, save the specimens, and export data in FCS 3.1 format for potential re-analysis in FlowJo software.

**Western Blot:** Total proteins were extracted from treated cells using RIPA buffer (P0013B) supplemented with protease inhibitors. Protein concentration was determined via BCA assay. Equal amounts of protein (20 µg per lane) were separated by SDS-PAGE and transferred onto PVDF membranes. After blocking with 5% non-fat milk for 1 h, the membranes were incubated overnight at 4°C with primary antibodies against flag (Cell Signaling Technology, 14793) and GAPDH (Proteintech, 60004-1). Following washing, membranes were probed with HRP-conjugated secondary antibody for 1 h at room temperature. The primary antibody is diluted at a ratio of 1:1000, and the secondary antibody is diluted at a ratio of 1:10000. Protein bands were visualized using an enhanced chemiluminescence (ECL) detection system.

**Immunofluorescence Staining:** Cells were fixed with paraformaldehyde and permeabilized for 20 minutes. After permeabilization, cells were washed with PBS three times (5 min per wash). Cells were blocked with blocking solution for 30 minutes. Cells were incubated with PAH antibody (Proteintech, 16347-1-AP) overnight at 4°C. Cells were washed three times with PBST (5 min per wash), followed by incubated with CoraLite594 Rabbit antibody (Proteintech, SA00013-4) 1h at room temperature. Primary antibody diluted at a ratio of 1:500. Secondary antibody diluted at a ratio of 1:400. Afterwards, the cells were washed another three times with PBST (5 min each). Coverslips were mounted using an aqueous mounting medium containing DAPI. Stained cells were examined using a fluorescence microscope (Life Technologies, EVOS FL Auto).

**Data Analysis:** Gel images of nested PCR products were captured using a gel imaging system (Uvitec), and contrast was adjusted using ImageJ software (v1.53). Raw qPCR data was analyzed using Bio-Rad CFX Maestro software (v2.3). FCS 3.1 files were further analyzed for gating strategy and result validation using FlowJo software (v10.8).

Statistical analyses were conducted with GraphPad Prism.

**Reference**

1.	Wilkinson, M.E., Frangieh, C.J., Macrae, R.K. and Zhang, F. (2023) Structure of the R2 non-LTR retrotransposon initiating target-primed reverse transcription. *Science*, **380**, 301-308.

2.	Eickbush, T.H. and Eickbush, D.G. (2015) Integration, Regulation, and Long-Term Stability of R2 Retrotransposons. *Microbiol Spectr*, **3**, MDNA3-0011-2014.

3.	Kuroki-Kami, A., Nichuguti, N., Yatabe, H., Mizuno, S., Kawamura, S. and Fujiwara, H. (2019) Targeted gene knockin in zebrafish using the 28S rDNA-specific non-LTR-retrotransposon R2Ol. *Mob DNA*, **10**, 23.

4.	Gorbunova, V., Seluanov, A., Mita, P., McKerrow, W., Fenyo, D., Boeke, J.D., Linker, S.B., Gage, F.H., Kreiling, J.A., Petrashen, A.P. *et al.* (2021) The role of retrotransposable elements in ageing and age-associated diseases. *Nature*, **596**, 43-53.
