# Supplementary material for: R2Tg retrotransposon mediated targeted integration of exogenous genes into the 28S rDNA locus in mammalian cell lines for PKU gene therapy
Source: Front Genome Ed. 2026 Sep 15;8:1893815. doi: 10.3389/fgeed.2026.1893815 (PMC13621248; doi:10.3389/fgeed.2026.1893815)
Supplement: Supplementary file 2 [file Supplementaryfile2.docx]

Supplementary of Original Data

Figure 2A

Figure 3A

Figure 2C
